# Emergence of carbapenem-resistant *Acinetobacter baumannii* as the major cause of ventilator-associated pneumonia in intensive care unit patients at an infectious disease hospital in southern Vietnam

**DOI:** 10.1099/jmm.0.076646-0

**Published:** 2014-10

**Authors:** Nguyen Thi Khanh Nhu, Nguyen Phu Huong Lan, James I. Campbell, Christopher M. Parry, Corinne Thompson, Ha Thanh Tuyen, Nguyen Van Minh Hoang, Pham Thi Thanh Tam, Vien Minh Le, Tran Vu Thieu Nga, Tran Do Hoang Nhu, Pham Van Minh, Nguyen Thi Thu Nga, Cao Thu Thuy, Le Thi Dung, Nguyen Thi Thu Yen, Nguyen Van Hao, Huynh Thi Loan, Lam Minh Yen, Ho Dang Trung Nghia, Tran Tinh Hien, Louise Thwaites, Guy Thwaites, Nguyen Van Vinh Chau, Stephen Baker

**Affiliations:** 1 The Hospital for Tropical Diseases, Wellcome Trust Major Overseas Programme, Oxford University Clinical Research Unit, Ho Chi Minh City, Vietnam; 2 School of Chemistry and Molecular Biosciences, The University of Queensland, Brisbane, Queensland, Australia; 3 The Hospital for Tropical Diseases, Ho Chi Minh City, Vietnam; 4 Centre for Tropical Medicine, Nuffield Department of Clinical Medicine, Oxford University, UK; 5 Clinical Sciences, Liverpool School of Tropical Medicine, Liverpool, UK; 6 Division of Infectious Diseases, Department of Medicine, University of California San Francisco, CA, USA; 7 Pham Ngoc Thach University of Medicine, Ho Chi Minh City, Vietnam; 8 The London School of Hygiene and Tropical Medicine, London, UK

## Abstract

Ventilator-associated pneumonia (VAP) is a serious healthcare-associated infection that affects up to 30 % of intubated and mechanically ventilated patients in intensive care units (ICUs) worldwide. The bacterial aetiology and corresponding antimicrobial susceptibility of VAP is highly variable, and can differ between countries, national provinces and even between different wards in the same hospital. We aimed to understand and document changes in the causative agents of VAP and their antimicrobial susceptibility profiles retrospectively over an 11 year period in a major infectious disease hospital in southern Vietnam. Our analysis outlined a significant shift from *Pseudomonas aeruginosa* to *Acinetobacter* spp. as the most prevalent bacteria isolated from quantitative tracheal aspirates in patients with VAP in this setting. Antimicrobial resistance was common across all bacterial species and we found a marked proportional annual increase in carbapenem-resistant *Acinetobacter* spp. over a 3 year period from 2008 (annual trend; odds ratio 1.656, *P* = 0.010). We further investigated the possible emergence of a carbapenem-resistant *Acinetobacter baumannii* clone by multiple-locus variable number tandem repeat analysis, finding a *bla*_OXA-23_-positive strain that was associated with an upsurge in the isolation of this pathogen. We additionally identified a single *bla*_NDM-1_-positive *A. baumannii* isolate. This work highlights the emergence of a carbapenem-resistant clone of *A. baumannii* and a worrying trend of antimicrobial resistance in the ICU of the Hospital for Tropical Diseases in Ho Chi Minh City, Vietnam.

## Introduction

Ventilator-associated pneumonia (VAP) is a common healthcare-associated infection among patients in intensive care units (ICUs) who have endotracheal intubation or a tracheostomy for mechanical ventilation. VAP is the most commonly acquired infection in ICUs worldwide, affecting an estimated 10–30 % of ventilated patients (Bantar *et al.*, 2008). The contribution of VAP to mortality in intubated patients is highly variable (Melsen *et al.*, 2009), ranging from 20 to 70 %, depending on the location and the study population (Davis, 2006; Rosenthal *et al.*, 2011). VAP prolongs the required duration of mechanical ventilation, increases the stay of the patient in an ICU and, when multidrug-resistant (MDR) organisms are present, increases the cost of treatment because of the need for expensive, non-first-line antimicrobials (van Nieuwenhoven *et al.*, 2004; Warren *et al.*, 2003).

There is a pronounced association between the aetiology of VAP and national economic development (Alp & Voss, 2006; Joseph *et al.*, 2010). In Europe and the USA, the principal organism associated with VAP is *Staphylococcus aureus* (Chastre *et al.*, 2014; Gaynes & Edwards, 2005; Joseph *et al.*, 2010; Lee *et al.*, 2013). However, in Asia and Latin America, the Gram-negative organisms *Pseudomonas aeruginosa*, *Acinetobacter* spp. and *Klebsiella pneumoniae* predominate (Chawla, 2008; Gales *et al.*, 2002). Whilst the geographical and temporal distribution of the infecting bacteria are variable, the causative agents of VAP are united in their ability to become resistant to a range of antimicrobials, presumably selected by the sustained use of the same antimicrobial(s) within the healthcare setting in which they circulate. Now, ICUs worldwide are highly accustomed to VAP caused by meticillin-resistant *Staphylococcus aureus*, MDR *P. aeruginosa* and highly resistant *A. baumannii* (Ayraud-Thévenot *et al.*, 2012; Gaynes & Edwards, 2005; NNIS System 2004; Tadros *et al.*, 2013). Studies in VAP patients in ICUs have demonstrated that the rapid initiation of antimicrobial therapy active against the infecting organism improves outcome (Koenig & Truwit, 2006). Correspondingly, a delay in appropriate antimicrobials is associated with a disease of increased severity and mortality (Morrow *et al.*, 2009). Surveillance of the aetiologies of VAP and their changing antimicrobial susceptibility profiles aids the development of appropriate management and treatment guidelines.

We aimed to describe and understand the aetiology and the changing antimicrobial susceptibility patterns of the bacteria causing VAP on an ICU ward in an infectious disease hospital in southern Vietnam. We retrospectively gathered microbial culture data from tracheal aspirates taken from patients with suspected VAP admitted onto the ICU ward at the Hospital for Tropical Diseases in Ho Chi Minh City, Vietnam between 2000 and 2010. We report a significant shift from *P. aeruginosa* to *Acinetobacter* spp. over the period of investigation, which was associated with the emergence of a clone of carbapenem-resistant *A. baumannii.*

## Methods

### 

#### Study site and patients.

This retrospective study was an analysis of routine laboratory data of diagnostic tests performed as standard-of-care. The data were anonymized before analysis and individual patient consent was not required. The site of the study was the Hospital for Tropical Diseases in Ho Chi Minh City in the south of Vietnam. The Hospital for Tropical Diseases is a 550-bed hospital that serves as a primary and secondary facility for the surrounding local population in Ho Chi Minh City, and a tertiary referral centre for infectious diseases for the 17 southern provinces of the country; it has a catchment population of ~40 million people. Nearly 70 % of the admissions to the Hospital for Tropical Diseases are resident in Ho Chi Minh City, with the remainder resident in the surrounding provinces. Patients without infectious diseases, including those with surgical requirements, tuberculosis, cancer, primary haematological disorders or immunosuppression [other than human immunodeficiency virus (HIV) infection], are generally referred to other healthcare settings in the city.

All data originated from patients admitted onto the ICU ward (a 30-bed ward for critically ill patients). The ward admits patients with a range of severe conditions, including septic shock, septicaemia, tetanus, acute respiratory failure, dengue haemorrhagic fever and those transferred from clinical wards requiring critical care for infectious diseases. The present study was performed with data recorded in the hospital database. The criteria for analysis were: admission to the ICU, intubated for mechanical ventilation (due to respiratory failure) with a tracheal aspirate collected because of suspected VAP. VAP was defined as pneumonia where the patient was on mechanical ventilation for >2 calendar days on the date of event, with the day of ventilator placement being day 1, *and* the ventilator was in place on the date of the event or the day before. If the patient was admitted or transferred to the ICU on a ventilator, the day of admission was considered as day 1. We analysed the distribution of bacteria in tracheal aspirates collected from those patients between 2000 and 2010. Patients known to be sero-positive for HIV were excluded.

#### Microbial identification and antimicrobial susceptibility testing.

The tracheal aspirates were collected according to the local standard operating procedures of the Hospital for Tropical Diseases. Patients were pre-oxygenated. Briefly, a standard 500 mm, 14-gauge tracheal aspiration catheter (Argyle Sherwood Medical) was attached to a 20 ml syringe filled with 20 ml sterile saline. The distal end was lubricated with sterile gel, introduced via the tracheostomy or endotracheal tube and advanced until significant resistance was encountered. The saline was instilled over 10–15 s, the tube then withdrawn 10–20 mm, the saline was immediately reaspirated and the catheter was then removed. Between 5 and 10 ml of fluid was recovered. No further aspiration was attempted during removal of the catheter to avoid contamination with tracheal secretions. Samples were transported to the microbiology laboratory, placed in a fridge at 4 °C and processed within 2 h of collection. The tracheal aspirate samples were examined by a Gram stain, and the aspirate fluid was diluted 1 : 1 with Sputasol (Oxoid) and incubated at 37 °C, with periodic agitation, until liquefaction. The sample was then diluted (1 : 1, 10^−1^ and 10^−2^) using Maximum Recovery Diluent (Oxoid), and 20 µl 1 : 1 diluent was inoculated onto blood agar and chocolate agar base plates. Additionally, 20 µl of the 10^−1^ and 10^−2^ dilutions was plated onto MacConkey media and blood agar base (all media were supplied by Oxoid Unipath). Inoculated media were incubated at 37 °C, and examined after 24 and 48 h of incubation. The threshold used to discriminate between infection and colonization was ≥1×10^5^ c.f.u. ml^−1^ (i.e. >20 colonies on either media from the 10^−2^ dilution). Colonies above this threshold were identified using an in-house bacteriological identification (biochemical short-set) kit and/or by API 20E and API 20NE kits following the manufacturer’s guidelines (bioMérieux).

Antimicrobial susceptibilities were tested at the time of isolation by the modified Bauer–Kirby disc diffusion method, as recommended by the Clinical and Laboratory Standards Institute guidelines (CLSI, 2012). Mueller–Hinton agar and antimicrobial discs were purchased from Unipath. *Escherichia coli* ATCC 25922 and *Staphylococcus aureus* ATCC 25923 were used as control strains for these assays. The inhibitory zone sizes were recorded and interpreted according to current Clinical and Laboratory Standards Institute breakpoint guidelines (CLSI, 2012).

Antimicrobial susceptibility testing was dependent on the bacterial species. For *Acinetobacter* spp., *Pseudomonas* spp. and *Enterobacteriaceae*, piperacillin/tazobactam (100/10 µg), imipenem (10 µg), amikacin (30 µg), ofloxacin (5 µg), ceftriaxone (30 µg) and ceftazidime (30 µg) were assayed throughout the study period; from 2005 onward, ticarcillin/clavulanic acid (75/10 µg) and cefepime (30 µg) were also assayed. The susceptibility and resistance breakpoints against imipenem and meropenem were ≤1 and ≥4 mg l^−1^, respectively. For *Staphylococcus* spp. and *Streptococcus* spp., co-trimoxazole (1.25/23.75 µg), penicillin (10 µg), vancomycin (30 µg), rifampicin (5 µg), gentamicin (10 µg) and meticillin (5 µg, from 2000 to 2004) or oxacillin (1 µg, from 2005 to 2010) were assayed.

#### Data collection and statistical analysis.

Patients admitted to the hospital ICU during the study period who had a tracheal aspirate performed for suspected VAP were included in this retrospective study. A member of the hospital staff routinely recorded the date of tracheal aspirate, the patient’s age and sex, the number of tracheal aspirates collected, the result of the culture (whether positive or negative and whether a significant result as a consequence of bacterial concentration), and the susceptibility of the isolate to commonly used antimicrobial agents. Data from these records were subsequently entered into Excel (Microsoft). Trends over the 11 year period, including the proportion of cultured isolates by year and the antimicrobial susceptibility patterns, were evaluated by logistic regression; odds ratios (ORs) are shown in per unit of time (per year). All statistical analysis was performed using Stata version 11 (StataCorp); *P*≤0.05 was considered statistically significant.

#### Molecular characterization of *Acinetobacter* spp.

DNA was extracted from the 34 *Acinetobacter* spp. isolate cultures from the tracheal aspirate samples taken in 2010 using the Wizard Genomic DNA Extraction kit (Promega). The quality and concentration of the DNA were assessed using a NanoDrop Bioanalyzer spectrophotometer (Thermo Scientific). Genomic DNA from all strains was standardized to a concentration of 25 ng µl^−1^ for further use.

The presence of four main groups of carbapenem-hydrolysing β-lactamases (carbapenemases) in carbapenem-resistant *Acinetobacter* spp. isolates was determined by using a previously described multiplex PCR method (Woodford *et al.*, 2006). PCR amplifications were performed using four primer pairs that were specific for *bla*_OXA-51_-like, *bla*_OXA-23_-like, *bla*_OXA-24_-like and *bla*_OXA-58_-like in 20 µl reaction volumes, containing 1 µl template genomic DNA, 0.2 µM each primer, 2 U *Taq* polymerase, 200 µM each deoxynucleoside triphosphate and 1.5 mM MgCl_2_ in 1× PCR buffer (all PCR reagents were supplied by Bioline). The selected isolates were also subjected to PCR amplification for the detection of the *bla*_NDM-1_ gene using a previously described method with an annealing temperature of 59 °C (Nordmann *et al.*, 2011). All PCR amplifications were visualized on 1 % agarose gels and photographed under UV light after staining with ethidium bromide; amplicons were compared with the predicted sizes and DNA sequenced using an ABI3700 sequencer as described below.

The 34 selected strains of *Acinetobacter* were genotyped using the MLVA [multiple-locus variable number tandem repeat (VNTR) analysis] method with some modifications (Pourcel *et al.*, 2011). Briefly, genomic DNA from each of the 34 *Acinetobacter* spp. was subjected to three multiplex PCR amplifications (in a total volume of 50 µl), in which the annealing temperature was set at 50 °C. The primers and PCR conditions for each of the multiplex PCR amplifications are listed in Table 1. The sizes of the amplicons at each locus were determined by capillary electrophoresis fragment analysis using an ABI 3130 XL capillary electrophoresis system (Applied Biosystems). For fragment analysis, 0.5 µl PCR amplicons were mixed with 9.25 µl Hi-di Formamide and 0.25 µl GeneScan 500 LIZ size standard (Applied Biosystems). The mixture was incubated for 3 min at 95 °C, chilled for 10 min and analysed. Resulting fragment analysis data were analysed using GeneMapper v4.0 (Applied Biosystems). Further, to determine the number of repeating units, the differently sized amplicons at each locus were sequenced. PCR amplicons were purified using a PCR purification kit (Qiagen) and sequenced using a BigDye Terminator Sequencing kit (Applied Biosystems). All data were analysed using a numeric coefficient in BioNumerics software (Applied Maths) and phylogenetic trees were reconstructed in Dendroscope v2.3.

**Table 1.** PCR amplification primers used for *Acinetobacter* spp. MLVA genotyping

PCR	Primer	Sequence (5′→3′)	Label
**Multiplex 1**	Abaum_3530_L	TGCAACCGGTATTCTAGGAAC	VIC
	Abaum_3530_R	CCTTGAACAACATCGATTACTGGA	
	Abaum_3002_L	GACTGAAGCAAGACTAAAACGT	FAM
	Abaum_3002_R	TCTGGGCAGCTTCTTCTTGAGC	
	Abaum_1988_L	GGCAAGGCATGCTCAAGGGCC	FAM
	Abaum_1988_R	CAGTAGACTGCTGGTTAATGAG	
**Multiplex 2**	Abaum_0845_L	AATTTTAATTCCAAATTGCTCC	FAM
	Abaum_0845_R	ACTTAAAATCGCATTTTTATCA	
	Abaum_2396_L	CAAGTCCAATCAACTCATGATG	VIC
	Abaum_2396_R	CTCCTGTAAGTGCTGTTCAGCC	
	Abaum_3468_L	CAGAAGTCACTGCATCTGCAAC	NED
	Abaum_3468_R	CGGTTGAAATTTTTTATAATGAAG	
**Multiplex 3**	Abaum_2240_L	CCCGCAGTACATCATGGTTC	FAM
	Abaum_2240_R	AGAACATGTATACGCAACTG	
	Abaum_0826_L	TGACTACTGAAACAGTTTTTG	FAM
	Abaum_0826_R	ATGATTGTACCGAGTAAAAGA	

## Results

### General observations

Over the 11 year study period, 515 individual patients admitted to the ICU at the Hospital for Tropical Diseases with suggestive features of VAP had at least one tracheal aspirate specimen taken for microbiological investigation. From these 515 patients with suspected VAP, microbiological data were retrieved from 492. In total, 372 patients (76 %) had a least one potential bacterial pathogen isolated at concentration of ≥10^5^ c.f.u. ml^−1^ in their tracheal aspirate sample. Of these patients, 277 (74 %) were male. The vast majority of the VAP patients were adults (median age 51 years, range 2–91 years), with children under the age of 16 years accounting for only 6 % of the total (this reflects the catchment of the hospital, as children are more likely to be admitted other healthcare settings in Ho Chi Minh City). As the paediatric patients constituted 6 % (30 patients) of the patient data for this study, analysis was performed globally and not stratified by age.

### Bacteriology of tracheal aspirates

Over the 11 year period, 765 tracheal aspirate samples were obtained from the 492 suspected VAP patients with available data. At least one potential bacterial pathogen was isolated at a concentration of ≥10^5^ c.f.u. ml^−1^ in 608 (80 %) tracheal aspirate samples from 372 patients. The total number of significant isolates was 696, indicating that the VAP patients were infected with a mean of 1.9 significant bacteria [i.e. 696 unique bacteria (distinguished by antimicrobial susceptibility profile if same species) were isolated from the tracheal aspirates of 372 patients].

**Fig. 1.**
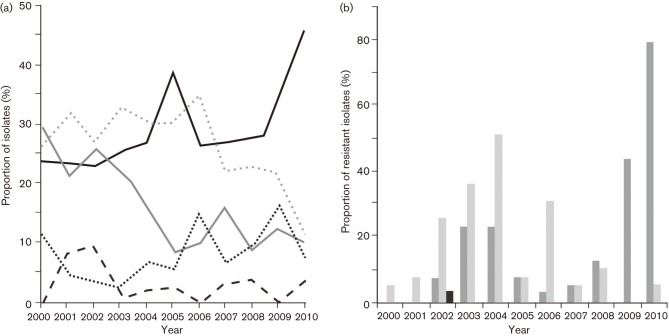
The changing aetiology of pathogens isolated from tracheal aspirates in ICU patients at the Hospital for Tropical Diseases in Ho Chi Minh City, Vietnam. (a) Proportion of selected bacterial isolates cultured from tracheal aspirates from 2000 to 2010. Black line, *Acinetobacter* spp.; grey line, *K. pneumoniae*; dashed black line, *Streptococcus pneumoniae*; dotted black line, *Staphylococcus* spp.; dotted grey line, *Pseudomonas aeruginosa.* (b) Histogram showing the proportion of *Acinetobacter* spp. (dark grey), *Pseudomonas aeruginosa* (light grey) and *K. pneumoniae* (black) cultured from tracheal aspirates showing resistance to imipenem from 2000 to 2010.

The bacterial pathogens isolated from significant tracheal aspirate specimens from the suspected VAP patients from 2000 to 2010, along with their corresponding time trends [ORs and *P* values for annual trend, with 95 % confidence intervals (CIs)], are outlined in Table 2. Gram-negative organisms predominated, accounting for 89 % of isolates (annual range 84–96 %). The most frequently cultured genus of bacteria were *Acinetobacter* spp. (30.4 %, *n* = 206), followed by *P. aeruginosa* (26.4 %, *n* = 186), *K. pneumoniae* (17 %, *n* = 118), *Staphylococcus* spp. (8.3 %, *n* = 53) and *Streptococcus pneumoniae* (3.1 %, *n* = 24) (Table 2). The annual prevalence rank of these aetiological agents changed several times between 2000 and 2007. In 2001, 2003, 2004 and 2006, the most prevalent isolates were *P. aeruginosa*, whereas *K. pneumonia* predominated in 2002 (Fig. 1a, Table 2). There was a subsequent shift in 2007 when *Acinetobacter* spp. became the most common group of pathogens isolated from patients with suspected VAP at the Hospital for Tropical Diseases (Fig. 1a) – a trend that continued until 2010.

**Table 2.** Bacterial pathogens isolated from intubated patients in ICU patients at the Hospital for Tropical Diseases in Ho Chi Minh City, Vietnam

Pathogen	2000	2001	2002	2003	2004	2005	2006	2007	2008	2009	2010	Mean	OR*	*P* value*	95 % CI
	[*n* (%)]	[*n* (%)]	[*n* (%)]	[*n* (%)]	[*n* (%)]	[*n* (%)]	[*n* (%)]	[*n* (%)]	[*n* (%)]	[*n* (%)]	[*n* (%)]	[*n* (%)]
*Acinetobacter* spp.	6	17	19	20	32	14	16	16	12	19	35	19	1.066	0.022	1.01–1.12
	(35.3)	(27.9)	(23.2)	(25.3)	(27.1)	(38.9)	(26.7)	(27.1)	(23.1)	(34.6)	(45.5)	(30.4)		
*K. pneumoniae*	5	13	24	18	19	3	6	11	4	7	8	11	0.873	<0.001	0.83–0.94
	(29.4)	(21.3)	(29.3)	(22.8)	(16.1)	(8.3)	(10.0)	(18.6)	(7.7)	(12.7)	(10.4)	(17.0)		
*P. aeruginosa*	4	20	22	26	36	11	21	13	12	12	9	17	0.917	0.004	0.86–0.97
	(23.5)	(32.8)	(26.8)	(32.9)	(30.5)	(30.6)	(35.0)	(22.0)	(23.1)	(21.8)	(11.7)	(26.4)		
*Streptococcus pneumoniae*	0	5	8	1	3	1	0	2	2	0	2	2	0.842	0.028	0.72–0.98
	(0.0)	(8.2)	(9.8)	(1.3)	(2.5)	(2.8)	(0.0)	(3.4)	(3.9)	(0.0)	(2.6)	(3.1)		
*Staphylococcus aureus*	2	3	3	2	8	2	9	4	5	9	6	5	1.117	0.021	1.02–1.23
	(11.8)	(4.9)	(3.7)	(2.5)	(6.8)	(5.6)	(15.0)	(6.8)	(9.6)	(16.6)	(7.8)	(8.3)		
Other Gram-negative bacteria†	0	3	6	10	20	5	8	13	17	8	17	11	1.207	<0.001	1.18–1.23
	(0.0)	(4.8)	(7.2)	(12)	(17)	(13.9)	(13.3)	(22.1)	(32.9)	(14.5)	(22.1)	(14.8)		

* Determined by logistic regression.

† Other Gram-negative bacteria include *Proteus* spp., *Alcaligenes* spp., *Citrobacter* spp., *Providencia* spp., *Serratia* spp., *Escherichia coli*, *Enterobacter* spp., *Burkholderia* spp., *Chryseobacterium* sp., *Haemophilus* spp. and *Stenotrophomonas maltophilia.*

In the initial 8 years of the time series, the annual proportion of *Acinetobacter* spp. was ~29 % of all isolates. This proportion dropped to 23 % in 2008, and then increased over the next 2 years to 35 and 45 % in 2009 and 2010, respectively. Over the entire 11 year period, the proportion of *Acinetobacter* spp. isolates increased by 6.6 % annually (OR 1.066, *P* = 0.022, 95 % CI 1.02–1.12). From 2008 to 2010, the proportional annual increase of *Acinetobacter* spp. was 10 times higher at 66 % (OR 1.656, *P* = 0.010, 95 % CI 1.13–2.43). In contrast, *P. aeruginosa* demonstrated a 9 % annual decrease (OR 0.917, *P* = 0.004, 95 % CI 0.86–0.97) over the entire study period. This pattern of declining *P. aeruginosa* was influenced by a marked drop from 2007 onwards, corresponding with a 25 % annual proportional reduction (OR 0.752, *P* = 0.003, 95 % CI 0.62–0.91) over the final 4 years of the study. *K. pneumoniae* was a significant VAP pathogen, yet was also associated with a significant annual decline.

Gram-positive pathogens causing VAP in ICU patients at the Hospital for Tropical Diseases were less common, and only *Streptococcus pneumoniae* and *Staphylococcus aureus* were longitudinally isolated and identified, comprising 3.13 and 8.25 % of all bacterial isolates, respectively. The proportion of *S. pneumonia* over the 11 years of study declined (OR 0.842, *P* = 0.028, 95 % CI 0.72–0.98), whilst *Staphylococcus aureus* isolates demonstrated a marginal proportional annual increase (OR 1.117, *P* = 0.021, 95 % CI 1.02–1.23) (Fig. 1a, Table 2).

### Antimicrobial susceptibility of isolated bacteria

The antimicrobial susceptibility profiles of the isolated potential pathogens exhibited substantial differences and were highly variable over the study period (Table 3). There was a decline in the proportion of *P. aeruginosa* and *K. pneumoniae* exhibiting antimicrobial resistance for all tested antimicrobials with the exception of piperacillin/tazobactam, ticarcillin/clavulanic acid and cefepime. *A. baumannii* demonstrated the most marked increase in resistance to the majority of antimicrobials tested. In 2010, ~86 % (30/35) of all isolated *Acinetobacter* spp. were resistant to all assayed antimicrobials, including aminoglycosides, cephalosporins and carbapenems (Table 3). Logistic regression demonstrated that there was a significant annual increase in the proportion of *Acinetobacter* spp. showing resistance to piperacillin/tazobactam and carbapenems, increasing by >50 % per year (Table 3). Furthermore, *Acinetobacter* spp. also exhibited an increase in resistance to the two additional antimicrobials that were used for susceptibility testing from 2005 onwards, with a proportional resistance increase of 100 % per year against cefepime and ticarcillin/clavulanic acid. The only exception to the increasing trend of *Acinetobacter* spp. antimicrobial resistance was ofloxacin, which displayed a proportional decrease by 11 % annually over the 11 years (OR 0.89, *P* = 0.012, 95 %CI 0.81–0.97). We additionally observed a substantial increase in the proportion of imipenem-resistant *Acinetobacter* spp. (Fig. 1b), increasing by 52 % annually over the study period. Imipenem was approved for empirical use for the treatment of VAP in this ICU in 2008. Imipenem resistance increased proportionally by more than three times annually between 2008 and 2010 (OR 3.27, *P* = 0.005, 95 %CI 1.43–7.49). This increasing resistance to carbapenems was not observed in other Gram-negative organisms. Only one imipenem-resistant *K. pneumoniae* was isolated during the study period (Fig. 1b).

**Table 3.** Percentage of bacterial isolates eliciting resistance to selected antimicrobials from tracheal aspirates in ICU patients at the Hospital for Tropical Diseases in Ho Chi Minh City, Vietnam

Antimicrobial	2000	2001	2002	2003	2004	2005	2006	2007	2008	2009	2010	OR	*P* value	95 %CI
Piperacillin/tazobactam	na	0.0	36.8	45.0	46.9	92.9	37.5	62.5	58.3	89.5	97.1	1.51	<0.001	1.33–1.71
Ticarcillin/clavulanic acid	na	na	na	na	na	42.9	25.0	62.5	66.7	84.2	97.1	2.09	<0.001	1.56–2.80
Ceftriaxone	100.0	70.6	89.5	95.0	93.8	92.9	6.3	68.8	100.0	100.0	97.1	1.07	0.304	0.94–1.20
Ceftazidime	83.3	64.7	73.7	65.0	68.8	78.6	12.5	62.5	75.0	94.7	97.1	1.15	0.008	1.04–1.27
Cefepime	na	na	na	na	na	28.6	50.0	37.5	58.3	94.7	97.1	2.25	<0.001	1.65–3.06
Imipenem	0.0	0.0	15.8	45.0	28.1	21.4	6.3	12.5	41.7	89.5	88.6	1.52	<0.001	1.35–1.72
Amikacin	33.3	52.9	79.0	60.0	59.4	85.7	50.0	50.0	75.0	73.7	94.3	1.16	0.003	1.05–1.29
Ofloxacin	83.3	47.1	36.8	65.0	59.4	71.4	50.0	43.8	58.3	47.4	22.9	0.89	0.012	0.81–0.97

na, Not applicable.

### Carbapenem-resistant *A. baumannii*

**Fig. 2.**
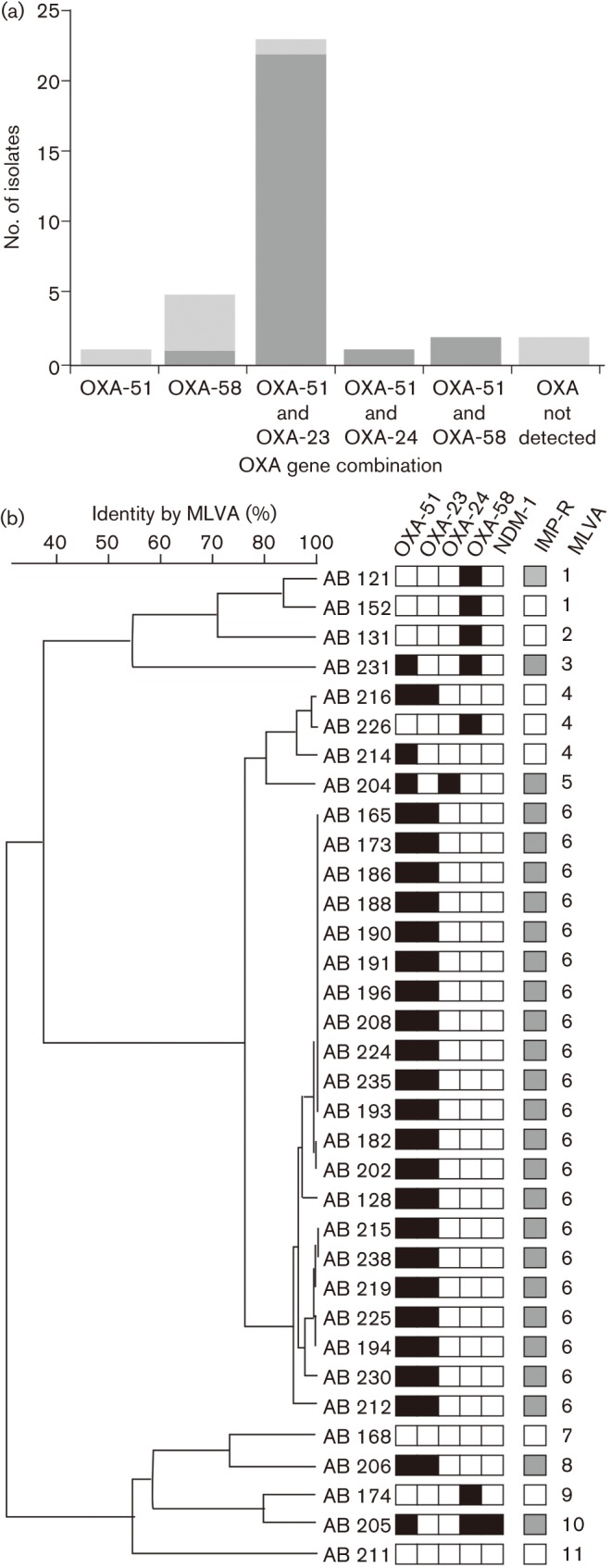
The distribution of OXA genes in 34 *Acinetobacter* spp. isolated from tracheal aspirates in ICU patients at the Hospital for Tropical Diseases in Ho Chi Minh City, Vietnam in 2010. (a) Histogram showing the number of *Acinetobacter* spp. (*n* = 34) producing PCR amplicons for OXA-51, OXA-58, OXA-23, OXA-24 and combinations thereof. Carbapenem-resistant isolates, dark grey; carbapenem-sensitive isolates, light grey. (b) Dendrogram created by MLVA using eight VNTR loci from 34 *Acinetobacter* spp. strains isolated in the ICU in 2010. The strain numbers are shown to the left of the dendrogram and the scale at the top of the diagram shows the percentage MLVA identity. Associated metadata data include the presence (black) or absence (white) of the OXA-51, OXA-58, OXA-23, OXA-24 and NDM-1 genes by PCR amplification, susceptibility to imipenem (IMP-R: susceptible, white; resistant, grey) and MLVA group (>90 % MLVA identity).

We hypothesized that the increase in imipenem-resistant *Acinetobacter* spp. was a result of selection of a specific *A. baumannii* clone containing several oxacillinase/carbapenemase genes. To test this hypothesis, we investigated 34 of the 35 available *Acinetobacter* spp. isolated from patients in 2010, screening them for *bla*_OXA-51_, *bla*_OXA-23_, *bla*_OXA-24_, *bla*_OXA-58_ and *bla*_NDM-1_, and by performing MLVA to investigate their relative genetic relationship. All five carbapenem-resistance-associated genes were detected in at least one strain and the majority of strains were PCR amplification-positive for more than one of the resistance loci, and 27 isolated were identified as *A. baumannii* by *bla*_OXA-51_ amplification (Fig. 2a). The most prevalent gene alongside *bla*_OXA-51_ was *bla*_OXA-23_, accounting for 68 % (23/34) of the *Acinetobacter* spp. isolates from 2010 (Fig. 2a). We noted an association between the presence of *bla*_OXA-51_ and *bla*_OXA-23_ and a MIC>32 µg ml^−1^ against imipenem (*P*<0.0001; Fisher’s exact test). Furthermore, we also detected the coexistence of *bla*_OXA-51_, *bla*_OXA-58_ and *bla*_NDM-1_ in one unique isolate (AB 205; MLVA10), which exhibited a MIC>32 µg ml^−1^ against imipenem, and high-level resistance to both third- and fourth-generation cephalosporins (Fig. 2). Of the *Acinetobacter* spp. that were susceptible to imipenem, these were mainly non-*A. baumannii*, and six out of eight isolates possessed at least one oxacillinase gene, the majority being PCR amplification-positive for *bla*_OXA-58_. With an arbitrary cutoff of 90 % identity over the eight selected VNTR loci, MLVA analysis classified the 34 isolates of *Acinetobacter* spp. into 11 discrete clusters; the *A. baumannii* isolates fell in seven of the clusters (Fig. 2b). The MLVA clusters were practically concordant with the presence of the various combinations of oxacillinase genes. Notably, one clone with >95 % identity (MLVA6) contained 21 out of 23 isolates harbouring both the *bla*_OXA-51_ and *bla*_OXA-23_ genes (MIC>32 µg ml^−1^) (Fig. 2b). The one exception was strain AB 216, which was PCR amplification-positive for both *bla*_OXA-51_ and *bla*_OXA-23_, but remained susceptible to imipenem.

## Discussion

Here, we show a series of significant changes in the prevalence of differing bacterial pathogens, and their corresponding antimicrobial susceptibility profiles, causing VAP over 11 years in one ICU ward in Ho Chi Minh City, Vietnam. We found that the most common pathogens causing VAP in this setting were *P. aeruginosa*, *K. pneumoniae*, *Acinetobacter* spp. and *Staphylococcus aureus*, which is broadly consistent with other locations. The striking finding was the emergence of *Acinetobacter* spp. as the most commonly isolated pathogen causing VAP in our ICU. Whilst the emergence of *Acinetobacter* spp. (specifically *A. baumannii*) has been observed in other locations in Asia, such as Malaysia, Pakistan, India and Thailand (Chawla, 2008; Lagamayo, 2008), the abruptness of the rise is of greatest concern. By 2010, *Acinetobacter* spp. were responsible for 46 % of all cases of VAP. This increased rate of isolation followed the introduction of imipenem as empirical treatment for VAP in 2008. Prior to 2008, imipenem-resistant *Acinetobacter* spp., except for a small increase in 2003/2004, comprised ~10 % of all *Acinetobacter* spp. By 2010, this proportion was almost 80 %. In contrast, the proportion of *P. aeruginosa* and *K. pneumoniae* isolations declined with a fall in resistance to imipenem. Only one imipenem-resistant *K. pneumoniae* was isolated over the whole study period, suggesting that carbapenem-resistant *K. pneumoniae* is not yet a problem in Vietnam, unlike other locations in Asia (Balm *et al.*, 2013; Hu *et al.*, 2013).

We hypothesized that a carbapenem-resistant *A. baumannii* clone had been introduced and then maintained on the ICU. Although our genotyping data were restricted to MLVA analysis and not whole-genome sequencing, which has been shown to be the gold standard in *Acinetobacter* spp. subtyping (Rolain *et al.*, 2013; Snitkin *et al.*, 2013), we were able to define several groups and the one *A. baumannii* major clone (>90 % MLVA identification) we named MLVA6. MLVA6 isolates were consistently resistant to imipenem and harboured the *bla*_OXA-23_ gene. The presence of the *bla*_OXA-23_ gene in carbapenem-resistant *A. baumannii* has been commonly reported in Asia and Latin America (Gales *et al.*, 2002; Mendes *et al.*, 2009). These genotyping data strongly support our hypothesis, and we further suggest that the MLVA6 clone was introduced onto the ICU before the empirical use of imipenem and then maintained by sustained usage. We additionally identified seven independent strains harbouring the *bla*_OXA-58_ gene, although the presence of this carbapenemase gene alone, or in combination with *bla*_OXA-51_, was generally not sufficient to induce carbapenem resistance. Notably, we identified one *A. baumannii* isolate that was PCR amplification (and sequence)-positive for *bla*_NDM-1_ (AB 205). Isolate AB 205 was resistant additionally to third- and fourth-generation cephalosporins, thus confirming the phenotype associated with *bla*_NDM-1_. The *bla*_NDM-1_ gene has been identified in Vietnam previously, but was associated with clinical and environmental isolates of *K. pneumoniae* (Hoang *et al.*, 2013; Isozumi *et al.*, 2012). We have not, as yet, isolated a *bla*_NDM-1_-positive *K. pneumoniae* clone on our ICU, but to our knowledge this is the first report of a *bla*_NDM-1_-positive *A. baumannii* from Vietnam.

The emergence of carbapenem-resistant *A. baumannii* in our ICU raises the question of antimicrobial therapy on the ward and plans for elimination. Following the emergence of carbapenem-resistant *A. baumannii* in our ICU, therapy for VAP has been modified to incorporate the polymyxin, colistin. Colistin is being used increasingly for the treatment of VAP and appears to be, at least currently, efficacious (Aydemir *et al.*, 2013; Jean & Hsueh, 2011). We predict that, similar to the emergence of resistance to carbapenems, colistin resistance will emerge here, have a similar impact on clonal selection (Rolain *et al.*, 2013) and leave us with effectively untreatable *A. baumannii* infections.

The observed changes in VAP pathogens likely reflect differing antimicrobial usage practices, and other demographic and economic shifts, that Vietnam has experienced over the last 10–15 years. Indeed, these demographic and economic changes have had other dramatic impacts on infectious disease dynamics in Vietnam over this period, as have been observed in enteric infections and bacteraemia in the same healthcare setting over a similar time period (Nga *et al.*, 2012; Vinh *et al.*, 2009). Although this changing disease epidemiology in Vietnam has resulted in some instances of bacteria causing severe infections replaced by pathogens that are more commonly found in developed countries and associated with less severe disease (Nga *et al.*, 2012), antimicrobial resistance and MDR bacterial pathogens and commensals have become commonplace (Holt *et al.*, 2013; Vien *et al.*, 2012). Access to antimicrobial agents is unrestricted in animals and humans in Vietnam, and we suggest that the overuse of, and sustained exposure to, antimicrobials is driving the selection of MDR bacteria with a substantial spread and prolonged circulation of antimicrobial-resistant organisms in the human population in the community and, as observed here, in healthcare settings.

This study has limitations. The data were retrieved from microbiological records and contemporaneous clinical data were not recorded. It is not possible to determine how many patients had a confirmed radiological pneumonia or simply tracheobronchitis, and we have no data on changes in disease outcome or the association of the duration of ventilation or hospital stay with the isolated pathogens. Prospective studies combining microbiological analysis with surveillance of patient outcomes and other end-points associated with disease severity are needed. However, a notable strength of this study is that the amalgamated data were collected over an extended period by the same clinical team using the same routine sample collection and microbiological methods. Our analysis was performed on data retrieved from a single ICU ward in an infectious disease hospital in southern Vietnam. Although it may not be wholly generalizable to other ICUs in Vietnam or across south-east Asia we suggest that our results indicate the broad consequence of the changing demographic structures and antimicrobial usage practices typical of these locations.

In conclusion, in this retrospective analysis of bacterial pathogens isolated from quantitative tracheal aspirates from ICU patients with suspected VAP in an infectious disease hospital in southern Vietnam over an 11 year period we found several shifts in the prevalence of specific VAP pathogens. In the latter part of the study the dominant pathogen was a carbapenem-resistant *A. baumannii* and strain analysis indicated the emergence of a carbapenem-resistant clone of *A. baumannii*, thus highlighting a worrying trend in the ICU of this infectious disease hospital. Future investigations should focus on optimizing the antimicrobial therapy of patients with VAP caused by these resistant *A. baumannii* strains.
